# Combined enzymatic hydrolysis and herbal extracts fortification to boost *in vitro* antioxidant activity of edible bird’s nest solution

**DOI:** 10.1016/j.chmed.2021.10.005

**Published:** 2021-10-07

**Authors:** Pei Ling Tang, Hooi Shin Goh, Swee Seng Sia

**Affiliations:** a Department of Bioscience, Faculty of Applied Sciences, Tunku Abdul Rahman University College, Setapak, 53300 Kuala Lumpur, Malaysia; b Nestlin Malaysia Sdn. Bhd., 3A, Jalan Payamas, Taman Industri Payamas, Tangkak 84900, Johor, Malaysia

**Keywords:** antioxidant, cinnamon twig, edible bird’s nest, enzymatic hydrolysis, ginger, mulberry leaves

## Abstract

**Objective:**

Edible bird’s nest (EBN) is a popular traditional tonic food in Chinese population for centuries. Malaysia is one of the main EBN suppliers in the world. This study aims to explore the best strategy to boost the antioxidant potential of EBN solution.

**Methods:**

In this study, the raw EBN (4%, mass to volume ratio) was initially enzymatic hydrolyzed using papain enzyme to produce EBN hydrolysate (EBNH), then spray-dried into powdered form. Next, 4% (mass to volume ratio) of EBNH powder was dissolved in ginger extract (GE), mulberry leaf extract (MLE) and cinnamon twig extract (CTE) to detect the changes of antioxidant activities, respectively.

**Results:**

Results obtained suggest that enzymatic hydrolysis significantly reduced the viscosity of 4% EBN solution from (68.12 ± 0.69) mPa·s to (7.84 ± 0.31) mPa·s. Besides, the total phenolic content (TPC), total flavonoid content (TFC), total soluble protein, DPPH scavenging activity and ferric reducing antioxidant power (FRAP) were substantially increased following EBN hydrolysis using papain enzyme. In addition, fortification with GE, MLE and CTE had further improved the TPC, TFC, DPPH scavenging activity and FRAP of the EBNH solution. Among the samples, MLE-EBNH solution showed the most superior antioxidant potential at (86.39 ± 1.66)% of DPPH scavenging activity and (19.79 ± 2.96) mmol/L FeSO_4_ of FRAP.

**Conclusion:**

This study proved that combined enzymatic hydrolysis and MLE fortification is the best strategy to produce EBN product with prominent *in vitro* antioxidant potential. This preliminary study provides new insight into the compatibility of EBN with different herbal extracts for future health food production.

## 1 Introduction

Edible bird’s nest (EBN) is made from the saliva of male swiftlet *Aerodramus* sp. or *Collocallia* sp*.* Malaysia is the world’s third largest EBN producer after Thailand and Indonesia. In Chinese literature, EBN is known as ‘Yan Wo’ which defined as the swift’s nest (Jamalluddin et al., 2019). It has been regarded as high-grade health food that symbolizes the status of ancient dignitaries as early as Tang Dynasty. The curative benefits of EBN were first reported in *Essential of Materia Medica* by Ang Wang of Qing Dynasty, while the monograph entitled *A Supplement to Compendium of Materia Medica* describes the EBN efficacy in boosting body health in detail. In the practice of traditional Chinese medicine (TCM), EBN has claimed to have therapeutic effect of moisturizing the lung, resolving phlegm and stop coughing. Thus, EBN demand, especially among the Chinese community is increasing since several decades (Dai et al., 2021). According to Jamalluddin et al. (2019), annual legal EBN imports in China was increased by about 8-fold in four years, from 22.5 tons in 2015 to 183.2 tons in 2019.

EBN is proven to contain 42%–63% of protein, 10.63%–27.26% of carbohydrate, 2.1%–7.3% of ash, and 0.14%–1.2% of lipid, depending on the species of the swiftlet. The main component in EBN is glycoprotein. Generally, carbohydrate of EBN composed of about 9% of sialic acid (mainly *N*-acetyl-4-*O*-acetylneuraminic acid), 7.2% galactosamine, 5.3% glucosamine, 16.9% galactose and 0.7% fucose (Ma & Liu, 2012). Glycoprotein and sialic acid are the two main nutrients that relate to the high medicinal values of EBN. EBN has been proven to display anti-influenza, anti-ageing, antioxidant, anti-inflammation and immunoregulation activities, inhibit hemagglutination, promote cell division and chondrocyte regeneration, increase dermal thickness, improve cognitive performance, bone strength and cardiovascular disorders (Dai et al., 2021, Yew et al., 2018, Haghani et al., 2016).

Noteworthy, enzymatic hydrolysis has been proven to improve the bioactivities of EBN. In the study by Guo et al. (2006), EBN hydrolyzed with pancreatin F enzyme was proven to exhibit a stronger inhibitory activity toward influenza viruses. The same enzymatic hydrolysis technique had been adopted by Matsukawa et al., 2011, Yew et al., 2014, Yew et al., 2018 in their study to evaluate the therapeutic effects of EBN hydrolysate on bone strength and Parkinson’s disease. Their research findings proved that pancreatin F-hydrolyzed EBN effectively improved bone strength and exerted neuroprotection in Parkinson’s disease. Besides, Ghassem et al. (2017) also reported that EBN hydrolysate produced two novel pentapeptides (Pro-Phe-His-Pro-Tyr and Leu-Leu-Gly-Asp-Pro) via pepsin-trypsin hydrolysis that exhibited excellent protective effect against hydrogen peroxide-induced oxidative cells damage. According to Zulkifli et al. (2019), the degree of hydrolysis, protein solubility, antioxidant, and hypoglycemic activity of EBN hydrolysate were influenced by the types of enzymes and time used in the hydrolysis process. EBN hydrolysate produced via alcalase hydrolysis contains more small molecular weight glycopeptides than those produced using papain and papaya juice. However, papain-hydrolysed EBN produced at a shorter hydrolysis time exhibited the highest DPPH scavenging activity. Furthermore, Yan et al. (2021b) claimed that EBN glycopeptides have a better digestibility, bioavailability and assimilation than glycoprotein for imparting potent biological activities, such as antihypertensive, antioxidant, immunomodulation, lipid-lowering, anti-inflammation, and anti-microbial activities.

Even though glycoprotein in EBN is proven to exhibit prominent bioactivities, EBN itself is not an excellent source of complete food protein (Ma & Liu, 2012). Up to recently, EBN still could not be claimed as a functional food due to the lack of concrete data from *in to vitro*, *in-vivo* and clinical research. Strategy to incorporate EBN into other health foods or addition of other functional ingredients into EBN product was recommended by Yan et al. (2021b) to enrich the nutrition value, as well as the market value of EBN product. According to Dai et al. (2021), pearl-EBN mixture was found to substantially reduce the rate of lipid peroxidation in brain tissues and improve superoxide dismutase level in red blood cells of rat, subsequently delay ageing. Besides, pearl-EBN mixture had also been proved to promote T-lymphocyte transformation and increased IgM level, hence enhance immunity. Nonetheless, similar research that reported on the investigation of the compatibility of EBN with other natural food ingredients is limited. Therefore, this study was carried out to explore the effect of incorporating different herbal extracts (ginger, mulberry leaves and cinnamon twig) in EBN hydrolysate produced via papain hydrolysis.

Ginger, mulberry leaves and cinnamon twig are the three commonly used remedies in traditional medicine practice since ancient times. Ginger is widely used to treat nausea, colds, headache, upset stomach, diarrhea, arthritis, rheumatism, relieve flatulence and aid digestion (Kiyama, 2020), whereas mulberry leaf is known to effectively relieve the symptoms of fever, sore throat, cough, protect the liver, improve eyesight, facilitate urination, and lower blood pressure (Gryn-Rynko et al., 2016). Besides, cinnamon twig has long been used as anti-pyretic, anti-rheumatic, anti-spasmodic, and stomachic (Lee & Lim, 2021). In ginger, both volatile oils and non-volatile constituents contribute to its sensory perception and health benefits. The main volatile components that contribute to the distinct aroma and taste of ginger are zingiberene, curcumene and farnesene, whereas gingerols, shogaols, paradols and zingerones are the components that contribute to the hot sensation. Present medicine and nutrition research proved that 6-gingerol and 6-shogaol are the main bioactive compounds in ginger/ginger extract that contribute to its anti-inflammation, antioxidant, immunomodulating and anti-cancer properties (Menon et al., 2021). Due to the substantial amount of protein content in mulberry leaves, it is widely used as a feedstock for silkworm and animal husbandry like sheep, cattle, and pig. Furthermore, high polyphenols (primarily quercetin, rutin, isoquercetin, astragalin), flavonoids (particularly moracins), iminosugar, glycoprotein, ecdysteroids, megastigmanes and 1-deoxynojirimycin (DNJ) content in mulberry leaf make it an excellent natural remedy for diabetes mellitus. In addition, mulberry leaf extract has also been proved to exhibit prominent hypolipidemic, antiatherogenic, anticancer, anti-bacterial, antioxidant, anti-inflammation, antidopaminergic and cardioprotective activities (Chan et al., 2020, Gryn-Rynko et al., 2016). Culinary and folk medicine uses of cinnamon has long been documented. Cinnamon is one of the most consumed spices that is also widely used in traditional medicine. The bioactive compounds profile of cinnamon differs among the parts and species. The main chemical constituents in cinnamon twig are *L*-bornyl acetate, caryophyllene oxide, γ-eudesmol, β-caryophyllene, T-cardinol, δ-cadinene, *trans*-β-elemenone, cadalene, and *trans*-cinnamaldehyde. These bioactive compounds had proven to exhibit excellent anti-inflammation, antioxidant, and anti-hyperglycemic activity. Among the bioactive compounds, cinnamaldehyde was claimed to impart the greatest bioactivity (Lee and Lim, 2021, Ribeiro-Santos et al., 2017, Lin et al., 2016). In view of the prominent nutrition benefits of ginger, mulberry leaf and cinnamon twig, these three ingredients were selected in this study.

This is a preliminary study to investigate the effect of enzymatic hydrolysis and compatibility of ginger extract (GE), mulberry leaf extract (MLE) and cinnamon twig extract (CTE) in edible bird’s nest. Initially, the edible bird’s nest (EBN) was enzymatically hydrolyzed using papain enzyme to produce EBN hydrolysate (EBNH). Then, EBNH was spray-dried into powdered form and dissolved in different extracts (GE, MLE and CTE). The effects of papain hydrolysis and herbal extracts fortification using GE, MLE and CTE on pH, viscosity, color, and antioxidant activity of EBN were determined. This study provides a basic insight into the development of innovative future functional foods.

## 2 Materials and methods

### 2.1 Materials

The edible bird’s nest (EBN) of *Aerodramus fuciphagus* used was supplied by Nestlin Malaysia Sdn. Bhd., Johor, Malaysia. The broken EBN fraction recovered from the cleaning procedure was used in this study. The cleaning procedures to remove inedible contaminants such as feathers and dirt were conducted in the production line of the company. The clean dried broken EBN pieces were ground into coarse granulates with a high-speed grinder. The commercial food grade papain enzyme used was supplied by Shaanxi Huikangyuan Biomedical Technology Co., Ltd (Shaanxi, China). Other chemicals used include sodium carbonate, Folin-Ciocalteau reagent, gallic acid, sodium nitrite, quercetin, aluminium chloride, sodium hydroxide, 1,1-diphenyl-2-picrylhydrazyl, 2,4,6-tripyridyl-s-triazine, ferric chloride, hydrochloric acid, glacial acetic acid, and ferrous sulphate were analytical grade with the brand name of Sigma (Missouri, U.S.), Merck (New Jersey, U.S.) and Chemsoln (Selangor, Malaysia).

### 2.2 Preparation of edible bird’s nest hydrolysate (EBNH) powder

EBNH was produced in pilot-scale at 50 L capacity using a stirred-tank chamber in Nestlin (M) Sdn. Bhd. Prior to hydrolysis, the clean dried EBN coarse granulates were soaked in reverse osmosis (RO) water at a ratio of 1:1 at room temperature for 15 min. Then, boiling water was added to make up the ratio of EBN-to-water at 1:50. Next, the mixture was boiled for 45 min to soften the EBN. After cooled down to 65 °C, the pH of the EBN suspension was adjusted to 6.5 using distilled vinegar. Next, about 2% of papain enzyme was added. Enzymatic hydrolysis of EBN proceeded for 2 h. At the end of the hydrolysis process, the hydrolysate was filtered through 5 µm and 1 µm polypropylene (PP) membranes. Results of our preliminary study indicate that EBNH contains approximately 3% free sialic acid and peptides with a molecular weight range of 3.5–125 kDa. Next, the produced EBNH was spray dried into powder using a spray dryer at inlet temperature of 210 °C, outlet temperature of 90 °C, atomizer speed of 370 rpm and feed flow rate of 8 mL/min.

### 2.3 Preparation of ginger, mulberry leaf, and cinnamon twig extracts

Prior to extraction, the outer skin of ginger was scrapped off, then washed with running tap water. The ginger, mulberry leaves and cinnamon twig were chopped into small pieces, then blended using a kitchen blender. Approximately 10% of ginger, mulberry leaves and cinnamon twig was boiled in RO water separately for 30 min to prepare ginger extract (GE), mulberry leaf extract (MLE) and cinnamon twig extract (CTE). The solid residues of ginger, mulberry leaves and cinnamon twig were removed through filtration using 2-layer cheese clothes.

### 2.4 Preparation of edible bird’s nest fortified solution

To prepare the EBN solution (control), 4% (mass to volume ratio) of EBN coarse granulates were soaked in RO water for 15 min, then boiled for 45 min. To prepare the EBNH solution, 4% of EBNH powder was dissolved in distilled water at room temperature. To prepare EBNH solution fortified with herbal extracts, 4% of EBNH powder was dissolved in GE, MLE and CTE extract, respectively at room temperature.

### 2.5 Physicochemical characterization of edible bird’s nest fortified solution

pH of herbal extracts, EBN, EBNH and fortified-EBNHs was measured by using a pH meter (Mettler Toledo, Switzerland). The shear viscosity of all samples was determined by using a rheometer with C-PTD 200 measuring geometry (MCR102, Anton Paar, Austria). The color of all samples was measured by using a LC100 spectrocolorimeter (Lovibond, United Kingdom). The color measurement was expressed as L* (lightness-darkness), a* (redness-greenness) and b* (yellowness-blueness). Total color difference (ΔE) between EBNH and fortified-EBNHs was calculated using formula:

$$\sqrt{{\left(L_{C}^{*}-L_{S}^{*}\right)}^{2}+{\left(a_{C}^{*}-a_{S}^{*}\right)}^{2}+{\left(b_{C}^{*}-b_{S}^{*}\right)}^{2}}$$

where subscript C indicates EBNH and subscript S indicates the respective samples.

### 2.6 Chemical characterization of edible bird’s nest solution

Total phenolic content (TPC), total flavonoid content (TFC) and total soluble protein content were used as the indicator to monitor the chemical changes in EBN solution after enzymatic hydrolysis and herbal extracts fortification.

TPC was determined according to the modified protocol by Aryal et al. (2019). Briefly, about 2.5 mL of 0.2 mol/L Folin-Ciocalteau reagent was mixed with 0.5 mL of sample/gallic acid standard solution (0.01–0.10 mg/mL). Then, 2 mL of 7.5% sodium carbonate was added into the mixture, followed by dark incubation at room temperature for 1 h. Absorbance of the mixture was read at a wavelength of 765 nm by using a spectrophotometer. TPC of the samples was determined by interpolating the gallic acid calibration curve.

TFC was determined according to procedures by Hao et al. (2018) with slight modifications. Briefly, 4 mL of distilled water was added into 1 mL of sample/quercetin standard solution (0.10–0.10 mg/mL), followed by the addition of 0.3 mL of 5% sodium nitrite solution. The mixture was incubated for 5 min at room temperature, then 0.3 mL of 10% aluminium chloride was added. After incubation for 6 min, 2 mL of 1 mol/L sodium hydroxide solution was added. Next, the total volume of the mixture was brought up to 5 mL with distilled water. Absorbance of the mixture was read at a wavelength of 510 nm by using a spectrophotometer. TFC of the samples was determined by interpolating the quercetin calibration curve.

Total soluble protein of all samples was determined using Bradford method as described by Gan, Chang, Mat Nasir, Babji, & Lim, 2020 with slight modifications. About 1 mL of sample/bovine serum albumin (BSA) standard solution (0.0025–2 mg/mL) was mixed with 5 mL of Bradford reagent (5-times dilution). After incubation at room temperature for 5 min, absorbance of the mixture was read at a wavelength of 595 nm by using a spectrophotometer. Total soluble protein content of the sample was determined by interpolating the BSA calibration curve.

### 2.7 Antioxidant activity of edible bird’s nest fortified solution

Antioxidant activity was determined based on DPPH scavenging activity and ferric reducing antioxidant power (FRAP). DPPH scavenging activity was determined according to procedures as described by Roy et al. (2010). About 0.1 mL of sample/water (control) was mixed with 3.9 mL of 0.06 mmol/L methanolic DPPH solution. The absorbance was read at 517 nm after dark incubation at room temperature for 30 min. DPPH scavenging activity was calculated as the percentage of scavenging activity using the formula:

$$\frac{A_{C}-A_{S}}{A_{C}}\times 100\%$$

where A_C_ represents the absorbance of control whereas A_S_ represents the absorbance of sample. FRAP was determined according to the modified protocol by Hao et al. (2018). Initially, FRAP reagent was prepared by mixing 300 mmol/L acetate buffer, 10 mmol/L 2,4,6-tris-(2-pyridyl)-s-triazine acidic solution (TPTZ) and 20 mmol/L ferric chloride hexahydrate solution at a ratio of 10:1:1. About 0.1 mL of sample/ferrous sulphate standard solution (10–100 µmol/L) was mixed with 3 mL of FRAP reagent, then incubated at 37 °C for 10 min. Absorbance of the mixture was measured at a wavelength of 593 nm by using a spectrophotometer.

### 2.8 Statistical analysis

All experiments were carried out in triplicates and the results were expressed as mean ± standard deviation. Statistical differences between the data were determined via one-way ANOVA and post-hoc Tukey test using SPSS software version 20 (IBM, New York, USA). Results with *P* < 0.05 was considered significant. Correlation between TPC, TFC, DPPH and FRAP was determined via Pearson correlation analysis with *P* < 0.05 as a significant correlation.

## 3 Results and discussion

### 3.1 Effect of enzymatic hydrolysis and herbal extracts fortification on pH, viscosity, and color of EBN solution

Table 1 compared the effects of enzymatic hydrolysis and herbal extract fortification using ginger extract (GE), mulberry leaf extract (MLE) and cinnamon twig extract (CTE) on the pH, viscosity, and color of EBN solution. Based on Table 1, pH of 4% EBNH solution had no significant difference (*P* > 0.05) with 4% EBN solution. During EBN hydrolysis, peptides with C-terminus (with –COOH group) and *N*-terminus (with –NH_2_ group) were released. At the neutral pH, carboxyl groups were deprotonated to form –COO^-^ groups while the amino groups were protonated to form –NH_3_^+^ groups. These reactions counteracting the majority of protons released, hence the pH of EBN and EBNH was no significant difference (Mat et al., 2018). Among the three extracts investigated in this study, CTE was the most acidic in nature, at which its pH was around 4.8. pH of GE and MLE was no significant difference from EBNH. Therefore, the preparation of EBNH solution using GE and MLE did not exert a significant effect on the pH of the solution. Compared to ginger and mulberry leaves, cinnamon contains a broader spectrum of organic acids (Ribeiro-Santos et al., 2017, Sanchez-Salcedo et al., 2016, Cheng et al., 2011). These organic acids contribute to the acidic pH of CTE. Thus, pH of EBNH solution was significantly reduced when fortified with CTE.

**Table 1:** Effect of enzymatic hydrolysis and herbal extracts fortification using GE, MLE and cinnamon twig extract (CTE on pH, viscosity, and color changes of EBN solution (mean ± SD, *n* = 3).

Samples	pH	Viscosity/(mPa·s)	Color			
			L*	a*	b*	ΔE
EBN	6.79 ± 0.11^a^	68.12 ± 0.69^a^	59.23 ± 0.21^a^	1.33 ± 0.21^e^	−1.40 ± 0.38^f^	N/A
EBNH	6.80 ± 0.07^a^	7.84 ± 0.31^b^	55.53 ± 0.12^b^	0.93 ± 0.06^f^	11.30 ± 0.91^e^	N/A
GE-EBNH	6.87 ± 0.06^a^	6.43 ± 0.33^c^	44.70 ± 1.51^c^	1.90 ± <0.00^e^	16.60 ± 0.47^d^	12.10 ± 1.55^c^
MLE-EBNH	6.82 ± 0.11^a^	7.82 ± 0.51^b^	25.23 ± 0.75^e^	11.27 ± 0.93^b^	22.80 ± 1.21^c^	33.75 ± 1.05^a^
CTE-EBNH	6.50 ± 0.03^b^	8.50 ± 0.40^b^	35.37 ± 1.40^d^	9.10 ± 0.62^c^	26.70 ± 1.92^b^	26.73 ± 0.49^b^
GE	6.82 ± 0.03^a^	1.16 ± 0.02^d^	54.23 ± 0.31^b^	1.13 ± 0.21^e^	12.10 ± 0.29^e^	N/A
MLE	6.77 ± 0.03^a^	1.29 ± 0.01^d^	34.20 ± 0.10^d^	13.70 ± 0.10^a^	38.90 ± 0.21^a^	N/A
CTE	4.79 ± 0.09^c^	1.13 ± 0.03^d^	46.33 ± 1.34^c^	4.53 ± 0.32^d^	28.30 ± 0.21^b^	N/A

Note: ^1^a-f: Different alphabets in the same column indicate there is significant difference (*P* < 0.05) among samples. ^2^EBN represents edible bird’s nest solution, EBNH represents edible bird’s nest hydrolysate solution, GE-EBNH represents edible bird’s nest hydrolysate solution containing ginger extract, MLE-EBNH represents edible bird’s nest hydrolysate solution containing mulberry leaf extract, CTE-EBNH represents edible bird’s nest hydrolysate solution containing cinnamon twig extract.

Based on results in Table 1, 4% EBN solution showed the highest viscosity at (68.12 ± 0.69) mPa·s. Enzymatic hydrolysis significantly reduced the viscosity by approximately 9 folds to (7.84 ± 0.31) mPa·s. Our preliminary result indicated that the 4% EBN solution used in this study contains a major fraction of 125–165 kDa protein, whereas the EBNH solution contains 3.5–125 kDa protein. This result was in accordance with the result reported by Yan et al. (2021a), whereby the range of molecular weight of protein and glycoprotein in 30-min double-boiled EBN was reported at 21.1–254.3 kDa and 42.0–148.8 kDa, respectively. Papain is a cysteine endopeptidase. It catalyzes the hydrolysis of peptide bonds through the deprotonation of the thiol group at its active site by the basic amino acid residue of the substrate (Trezza et al., 2020, Singh et al., 2019). Ali et al. (2019) reported that the glycoprotein of EBN contains approximately 15% basic amino acids, which included 7.18% of arginine, 4.29% of histidine and 3.54% of lysine. Therefore, papain enzyme was expected to cleave the peptide bonds in the glycoprotein of EBN at the site of these amino acids to release glycopeptides. Furthermore, Singh et al. (2019) also claimed that papain enzyme is efficient in hydrolyzing high molecular weight protein. Effective protein hydrolysis using papain to produce bioactive peptides was also reported in other animal proteins, such as Chinese sturgeon (Noman et al., 2018), chicken feet collagen (Dhakal et al., 2018), fisheries residues (Tacias-Pascacio et al., 2021), as well as EBN (Zulkifli et al., 2019). Viscosity reduction is one of the indicators of protein hydrolysis. Viscosity of protein solution depends on the molecular size, aggregation state and molecular structure of the protein. Intermolecular interactions such as electrostatic interactions and disulfide bonds influenced the apparent viscosity of a protein solution. Large molecular size and high protein–protein aggregation contribute to high viscosity in protein solution (Averina et al., 2021). This explains the low viscosity of EBNH solution in this study.

Besides, results in Table 1 also showed that fortification of EBNH solution with MLE and CTE did not significantly (*P* > 0.05) affect its viscosity. Nevertheless, fortification with GE significantly reduced the viscosity of GE-EBNH by about 18% to (6.43 ± 0.33) mPa·s. Covalent interactions between phenolics and protein/peptide molecules modify the protein/peptide conformation, and hence its physicochemical and bioactive properties (Yan et al., 2021b). The magnitude of phenolic-protein interactions is dependent on the amino acids composition and sequence of a protein/peptide molecule and the diversity of phenolics. The surface hydrophobicity of a protein/peptide will reduce when hydrophobic phenolic compounds are covalently bound to the protein/peptide molecule (Yan et al., 2020). According to Ali et al. (2019), glycoprotein of EBN contains approximately 43% hydrophobic amino acids. Meanwhile, gingerol and shogaol are the main hydrophobic phytochemicals in ginger (Menon et al., 2021). Perhaps, the interaction between gingerol/shogaol with the hydrophobic residues of the EBNH glycopeptides is the factor that led to the slight reduction of viscosity of GE-EBNH solution. Considering the structural diversity of phenolic compounds in different extracts, the interaction behavior of protein–phenolic complexes could be more complex (Yan et al., 2020). This explains why the effect of GE, MLE and CTE on the viscosity of EBNH solution was different.

Based on Table 1, L* and a* values of EBN solution were significantly higher than EBNH solution, whereas b* of EBN solution was lower than EBNH solution. This result unveils that EBNH solution was slightly dull and more yellowish in color than the EBN solution. The minor color change was likely due to the high temperature exerted to the EBNH during spray drying process. In the study by Gan, Chang, Mat Nasir, Babji, & Lim, 2020, drying temperature was proven to exert significant effect on the color of EBN powder. High temperature drying causes cameralization reaction that increases the yellowness of the product. However, the exposure of EBN to high temperature during spray drying was short, hence the color change was small. In addition, relatively higher light scattering in EBNH solution than EBN solution might also be the reason for a lower L* value in EBNH solution. According to Cheng et al. (2018), particle size of compounds in a solution will influence the magnitude of wavelength scattering and reflectance, eventually contribute to color difference. Among the samples, fortification of EBNH with MLE (MLE-EBNH) caused the most perceivable color change with ΔE at (33.75 ± 1.05)°. The color of MLE-EBNH solution turned light orangish-brown after fortified with MLE. According to Table 1, MLE had the highest a* (redness) and b* (yellowness) value, and the lowest L* (lightness) value. The MLE solution was appeared orangish-brown in color with L* = (34.20 ± 0.10)°, a* = (13.70 ± 0.10)° and b* = (38.90 ± 0.2)°. The color change of EBNH solution from pale yellow to orangish-brown in MLE-EBNH solution was mainly contributed by the pigments in MLE. The orangish-brown color of MLE was believed contributed by the color pigments formed following the action of endogenous oxidative enzymes (polyphenol oxidase and peroxidase) and the degradation of chlorophyll in the MLE during the preparation step. During blending, the cell wall of fresh mulberry leaves was broken down, hence causing the release of endogenous oxidative enzymes into the solution, subsequently accelerate the rate of oxidation. However, incorporating EBNH into MLE had significantly reduced L*, a* and b* values of MLE-EBNH as compared to the color of MLE. As a comparison with CTE-EBNH, GE-EBNH had a light yellow color, whereby its b* value was lower and L* value was higher than CTE-EBNH. The influence of GE on the color of GE-EBNH solution was weak due to its light color in nature. The L* and b* values of GE were not significantly different (*P* > 0.05) with the EBNH solution. However, the color of GE-EBNH was duller and more yellowish in comparison to EBNH solution. Different pigment compounds may interact differently with the glycoprotein, hence changing the scattering and reflection properties of the wavelength and subsequently cause color change. Cheng et al. (2018) claimed that chemical composition, temperature, pH, molecular size, and their interactions significantly influenced the wavelength reflectance, and thus the color of a product.

### 3.2 Effect of enzymatic hydrolysis and herbal extracts fortification on the chemical characteristics of EBN solution

Table 2 presented the effect of enzymatic hydrolysis and herbal extract fortification using GE, MLE and CTE on the total phenolic (TPC), total flavonoid (TFC) and total soluble protein of EBN solution. Based on Table 2, TPC in 4% EBN solution was very low at 0.02 ± <0.00 mg GAE·mL^−1^, whereas TFC and total soluble protein content were too low to be quantified. However, TPC, TFC and total soluble protein were substantially increased to (0.83 ± 0.01) mg GAE· mL^−1^, 0.08 ± <0.00 mg QE·mL^−1^ and (10.50 ± 1.11) mg/mL respectively in 4% EBNH solution after 2 h papain hydrolysis. According to Ali et al. (2019), glycoprotein of EBN contains approximately 5.4% of tyrosine, which was the phenol derivative. Papain hydrolysis unfolded and cleaved the EBN glycoprotein into glycopeptides, which expose more tyrosine for detection in the analysis. This might be the reason for the increase of TPC in EBNH solution. Moreover, total soluble protein content was also increased following the breakdown of large molecular size glycoprotein by the action of papain enzyme. Small molecular size peptide has better solubility than the large molecular size protein. During enzymatic hydrolysis, more hydrophobic amino acids were exposed. When the catalysis is progressed, hydrophobic interactions between the peptide chains took place, which in turn, reduced the surface hydrophobicity. Besides, the formation of carboxylic acid and amine groups in the terminal amino acid residues increased the hydrophilicity of EBN glycopeptide (Cotabarren et al., 2019, Tang et al., 2019, Noman et al., 2018). This explains the higher total soluble protein content in EBNH solution than in EBN solution.

**Table 2:** Effect of enzymatic hydrolysis and herbal extracts fortification using GE, MLE and CTE on total phenolic, total flavonoid and total soluble protein content of EBN solution (mean ± SD, *n* = 3).

Samples	Total phenolic content/(mg GAE·mL^−1^)	Total flavonoid content/(mg QE·mL^−1^)	Total soluble protein/(mg·mL^−1^)
EBN	0.02 ± <0.00^f^	<0.00	<0.00
EBNH	0.83 ± 0.01^c^	0.08 ± <0.00^e^	10.50 ± 1.11^b^
GE-EBNH	0.88 ± 0.02^b^	0.20 ± 0.04^d^	8.71 ± 0.46^c^
MLE-EBNH	1.38 ± 0.02^a^	0.76 ± 0.20^a^	<0.00
CTE-EBNH	0.89 ± 0.02^b^	0.59 ± 0.03^b^	15.48 ± 1.46^a^
GE	0.09 ± <0.00^e^	0.09 ± <0.00^e^	0.27 ± 0.09^d^
MLE	0.80 ± 0.01^c^	0.53 ± 0.05^b^	0.21 ± 0.05^d^
CTE	0.27 ± <0.00^d^	0.37 ± 0.02^c^	0.07 ± 0.01^e^

Note: ^1^a-e: Different alphabets in the same column indicate there is significant difference (*P* < 0.05) among samples. ^2^EBN represents edible bird’s nest solution, EBNH represents edible bird’s nest hydrolysate solution, GE-EBNH represents edible bird’s nest hydrolysate solution containing ginger extract, MLE-EBNH represents edible bird’s nest hydrolysate solution containing mulberry leaf extract, CTE-EBNH represents edible bird’s nest hydrolysate solution containing cinnamon twig extract.

Among the extracts, MLE was proven to contain the highest TPC [(0.80 ± 0.01) mg GAE·mL^−1^] and TFC [(0.53 ± 0.05) mg QE·mL^−1^]. Thus, fortification of EBNH with MLE produced MLE-EBNH solution that contained the highest TPC [(1.38 ± 0.02) mg GAE·mL^−1^] and TFC [(0.76 ± 0.20) mg QE·mL^−1^]. Among the fortified EBNH solutions, GE-EBNH solution contained the lowest TFC [(0.20 ± 0.04) mg QE·mL^−1^], but its TPC was no significant difference with CTE-EBNH solution. The TPC and TFC of fortified EBNH solutions were found no significant difference with their sum content in EBNH solution and the respective extracts. This finding postulates that glycopeptides of EBNH will not reduce the availability of polyphenols and flavonoids in the extracts. According to Liu et al. (2017), hydrophobic interaction and hydrogen bonding between polyphenols and proteins facilitate the formation of the stable noncovalent complex. However, the polyphenol-protein complex formation is dependent on the structural characteristics of the protein and polyphenols. The bioavailability of polyphenols will be reduced if more polyphenol-protein complexes are formed. In the study by Hernandez-Jabalera et al. (2015), peptide-phenolic interaction diminished the *in-vitro* antioxidant capacity of rapeseed protein hydrolysate. Results in this study propose that combined EBNH and herbal extracts (GE, MLE and CTE) will be an effective approach to enhance the nutrition value of EBN product.

Besides, the results in Table 2 indicated that all extracts contained low soluble protein content. However, fortification of EBNH solution with GE and MLE significantly reduced its total soluble protein content, whereas CTE increased the total soluble protein content in CTE-EBNH solution. Surprisingly, total soluble protein in MLE-EBNH solution was too low to be detected even though its viscosity was no significant difference (*P* > 0.05) with the EBNH solution. Furthermore, results in Table 2 also suggested that the reduction of total soluble protein content in GE-EBNH was associated with the reduction of viscosity. In contrast, there was no significant change in viscosity of CTE-EBNH compared to EBNH solution (Table 1), even though the total soluble protein content in CTE-EBNH solution was increased by approximately 47% to (15.48 ± 1.46) mg/mL after CTE was added. This finding unveils that phytochemicals in GE, MLE and CTE influenced the attractive and repulsive interactions between the glycopeptide molecules in a distinct manner. Additives added into a protein solution modify the protein–protein and protein-solute interactions hence influenced the solubility of the protein, as well as the viscosity of the protein solution. Interaction of phytochemicals with protein molecules changes the net charge of the protein, eventually influenced the ratio of hydrophobicity and hydrophilicity of the protein. Protein with high solubility may not always be associated with low viscosity and vice versa. Both viscosity and solubility of a protein molecule are contributed by complex interactions (Tang et al., 2019, Hong et al., 2018, Hernández-Jabalera et al., 2015). Moreover, the method of soluble protein quantification is also one of the factors that influenced the results. In Bradford assay, Coomasive Brilliant Blue G-250 dye was bound to arginine, histidine, phenylalanine, tryptophan, and tyrosine residues of the protein, and changes the absorbance to 595 nm. However, the accuracy of the method will be influenced by the accessibility of the dye to the protein molecule. Any additive that hinders the dye-protein binding causes a high degree of underestimation (Khramtsov et al., 2021). Thus, undetected total soluble protein content in MLE-EBNH solution might be due to the interference of high phytochemicals in MLE-EBNH solution hindered the binding of Coomasive Brilliant Blue G-250 dye to glycopeptides of EBN.

### 3.3 Effect of enzymatic hydrolysis and herbal extracts fortification on antioxidant activity of EBN solution

Table 3 showed the effect of enzymatic hydrolysis and fortification using GE, MLE and CTE on the DPPH scavenging activity and ferric reducing antioxidant power (FRAP) of edible bird’s nest solution. Based on Table 3, DPPH scavenging activity [(8.98 ± 0.51)%] and FRAP [(0.65 ± 0.03) mmol/L FeSO_4_] of EBN solution were the lowest. Papain hydrolysis increased the DPPH scavenging activity and FRAP by almost 2-fold [(17.13 ± 2.18)%] and 4-fold [(2.65 ± 0.22) mmol/L FeSO_4_] respectively in the EBNH solution. Among the 18 amino acids detected in EBN, cysteine, methionine, tyrosine, tryptophan, phenylalanine, and histidine are the amino acids that exhibit antioxidant activity. Antioxidant activity exhibited by these amino acids probably contributed by its sulfhydryl and aromatic groups. In the well-folded native protein molecule, these hydrophobic amino acids, except histidine mostly embedded in the protein structure. During papain hydrolysis, the glycoprotein was unfolded and cleaved into glycopeptides. Thus, these amino acids were exposed. This explains the higher antioxidant activity of EBNH than EBN (Ali et al., 2019, Gan, Chang, Mat Nasir, Babji, & Lim, 2020, Quek et al., 2018).

**Table 3:** Effect of enzymatic hydrolysis and herbal extracts fortification using GE, MLE and CTE on DPPH scavenging activity and ferric reducing antioxidant power (FRAP) ofEBN solution (mean ± SD, *n* = 3).

Samples	DPPH scavenging activity/%	Ferric reducing antioxidant power/(mmol·L^−1^ FeSO_4_)
EBN	8.98 ± 0.51^f^	0.65 ± 0.03^b^
EBNH	17.13 ± 2.18^e^	2.65 ± 0.22^b^
GE-EBNH	47.83 ± 0.56^d^	3.73 ± 0.86^b^
MLE-EBNH	86.39 ± 1.66^a^	19.79 ± 2.96^a^
CTE-EBNH	75.96 ± 0.83^b^	5.47 ± 0.26^b^
GE	56.59 ± 0.89^c^	3.01 ± 0.39^b^
MLE	87.79 ± 1.35^a^	22.29 ± 4.23^a^
CTE	86.46 ± 0.84^a^	5.51 ± 0.59^b^

Note: ^1^a–f: Different alphabets in the same column indicate there is significant difference (*P* < 0.05) between samples. ^2^EBN represents edible bird’s nest solution, EBNH represents edible bird’s nest hydrolysate solution, GE-EBNH represents edible bird’s nest hydrolysate solution containing ginger extract, MLE-EBNH represents edible bird’s nest hydrolysate solution containing mulberry leaf extract, CTE-EBNH represents edible bird’s nest hydrolysate solution containing cinnamon twig extract.

Besides, the results also indicate that MLE and CTE exhibited the highest DPPH scavenging activity (86%–88% inhibition), followed by GE [(56.59 ± 0.89) % inhibition]. In addition, FRAP of CTE [(5.51 ± 0.59) mmol/L FeSO_4_] was found about 75% lower than MLE [(22.29 ± 4.23) mmol/L FeSO_4_] and GE had the lowest FRAP [(3.01 ± 0.39) mmol/L FeSO_4_]. DPPH and FRAP assays measured antioxidant potential in different mechanisms. DPPH assay measures the ability of the extract to donate protons to neutralize the reactive free radicals, whereas FRAP measures the ability of the extract to donate electrons to reduce ferric ions to ferrous ions (Gan, Chang, Mat Nasir, Babji, & Lim, 2020). Among the fortified-EBNH samples, MLE-EBNH exhibited the highest DPPH scavenging activity [(86.39 ± 1.66)% inhibition] and FRAP [(19.79 ± 2.96 mmol/L FeSO_4_]. The DPPH scavenging activity and FRAP of MLE-EBNH were no significant difference with MLE. Results of Pearson correlation analysis reveal that DPPH scavenging activity of fortified-EBNH solutions and extracts was strongly correlated with its TFC (*r* = 0.843 at *P* < 0.01), whereas FRAP was positively correlated with its TPC (*r* = 0.592 at *P* < 0.01) and TFC (*r* = 0.767 at *P* < 0.01). This finding recommends that glycoprotein in EBNH did not bound or form crosslink with polyphenols and flavonoids in MLE when both components were combined. Antioxidant activity of MLE-EBNH solution was mainly contributed by mainly the TPC and TFC of MLE. However, it was found that the DPPH scavenging activity of GE-EBNH and CTE-EBNH solutions was significantly lower than GE and CTE, respectively. DPPH scavenging activity of GE-EBNH and CTE-EBNH was about 15% and 12% lower than GE and CTE, respectively. Nevertheless, FRAP of GE-EBNH and CTE-EBNH was no significant difference (*P* > 0.05) with GE and CTE, respectively. This finding proposes that glycoprotein in EBNH may interact with functional groups of some flavonoids in GE and CTE, hence reduced the DPPH scavenging activity in GE-EBNH and CTE-EBNH. Antioxidant activity of protein hydrolysate is strongly correlated with the phenolic-protein interaction (Hernandez-Jabalera et al., 2015). Due to the minimum interactions between phenolics and flavonoids with glycopeptides, antioxidant activity of the fortified-EBNH solutions was either no significant or slightly lower than its respective extract.

## 4 Conclusion

This study concluded that papain hydrolysis was the best technique to improve TPC, TFC, total soluble protein content and antioxidant potential of edible bird’s nest. Fortification of EBNH with ginger, mulberry leaf and cinnamon twig extracts further increased the TPC, TFC and antioxidant activity. Among the fortified samples, edible bird’s nest hydrolysate fortified with mulberry leaf extract (MLE-EBNH) contained the highest TPC, TFC and antioxidant activity, even though the mulberry leaf extract had significantly affected the color of the solution. In conclusion, MLE-EBNH was the best product that contains the nutrition benefits of both mulberry leaf (high phytochemicals) and EBN (high glycoprotein).

## Declaration of Competing Interest

The authors declare that they have no known competing financial interests or personal relationships that could have appeared to influence the work reported in this paper.
